# Association of sex steroid hormones and new bone formation rate after iliac onlay grafting: a prospective clinical pilot study

**DOI:** 10.1186/s40729-022-00447-x

**Published:** 2022-11-15

**Authors:** Victoria Constanze Landwehr, Tobias Fretwurst, Julia Heinen, Kirstin Vach, Katja Nelson, Susanne Nahles, Gerhard Iglhaut

**Affiliations:** 1 Department of Oral and Maxillofacial Surgery, Center for Dental Medicine, Medical Center – University of Freiburg, Faculty of Medicine – University of Freiburg, Hugstetter Straße 55, 79106 Freiburg, Germany; 2 Department of Oral and Maxillofacial Surgery, Berlin Institute of Health, Corporate Member of Freie Universität Berlin, Charité – Universitätsmedizin Berlin, Humboldt-Universität Zu Berlin, Augustenburger Platz 1, 13353 Berlin, Germany; 3 Institute of Medical Biometry and Statistics, Faculty of Medicine and Medical Center, University of Freiburg, Hebelstraße 11, 79104 Freiburg, Germany

**Keywords:** Augmentation, Body mass index, Dental implant, Estradiol, Testosterone

## Abstract

**Purpose:**

The present prospective study evaluates the association between new bone formation rate in the iliac onlay graft and sex steroid hormone serum levels.

**Methods:**

A total of 15 partially or completely edentulous postmenopausal females and 9 males with less than 5 mm height of the remaining alveolar bone underwent iliac onlay grafting followed by dental implant placement using a two-stage approach. Sex hormone binding globulin and 17β-estradiol serum levels were investigated by electrochemiluminescence immunoassay, while total testosterone level was analyzed using radioimmunoassay. At the time of implant placement, 12 weeks after grafting, bone biopsies were obtained and analyzed histomorphometrically. Statistical analysis was performed using linear mixed models.

**Results:**

Grafting procedure was successfully performed in all patients. The mean new bone formation rate was 32.5% (116 samples). In men the mean new bone formation rate (38.1%) was significantly higher (*p* < 0.01) than in women (27.6%). Independent of gender 17β-estradiol and testosterone were positively associated to overall new bone formation rate, albeit a significant influence was only seen for 17β-estradiol in men (*p* = 0.020). Sex hormone binding globulin had no influence on new bone formation rate (*p* = 0.897). There was no significant association between new bone formation rate and age (*p* = 0.353) or new bone formation rate and body mass index (*p* = 0.248).

**Conclusion:**

Positive association of 17ß-estradiol as well as testosterone with new bone formation rate after iliac onlay grafting indicates a role of sex steroid hormones in alveolar bone regeneration, although the observed influence was only significant for 17ß-estradiol in men.

**Graphical Abstract:**

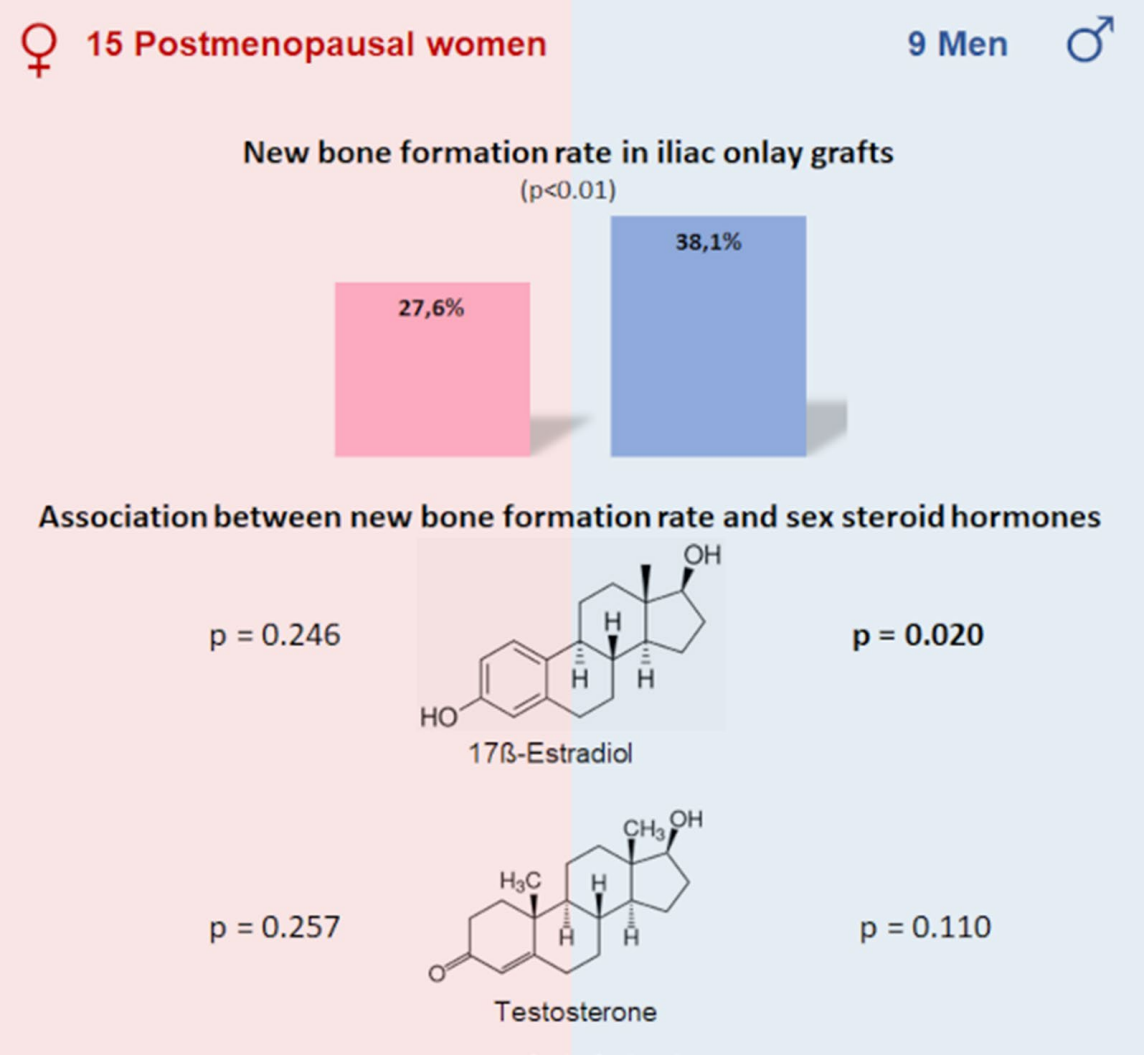

**Supplementary Information:**

The online version contains supplementary material available at 10.1186/s40729-022-00447-x.

## Background

Resorbed alveolar ridges after tooth loss can successfully be treated with diverse augmentation procedures in combination with dental implant placement [1]. In case of severely atrophied jawbone, the success of autologous avascular iliac bone grafting has been well documented [2–5]. Clinical and histomorphometric studies show sufficient revascularization for secure dental implant placement after a healing period of 3 to 4 months [2, 3]. Long-term implant survival rates following a two-stage augmentation procedure with cortico-cancellous iliac bone grafts can be up to 99% after a 5-year observation period [4, 5]. The rate of new bone formation (NBF) in iliac onlay grafts shows pronounced interindividual differences varying from 3 to 98% within the same healing time [2, 3]. Bone metabolism is a multifactorial process which seems to be influenced by nutrition, physical activity, body mass index (BMI), age, medication and sex [6–10]. Sex steroid hormones have been shown to affect bone metabolism in vivo as well as in vitro [11, 12].

Bone maturation and bone homeostasis is known to be influenced by estrogen and androgen sex steroid hormones [12]; as the respective hormone receptor has been detected in cells resident in the bone including osteoblasts, osteocytes, osteoclasts and bone marrow stromal cells [13–15]. Being lipids, the majority of sex steroid hormones are transported bound to a protein via blood, while only 1–2% are circulating unbound (free) [16]. Among other physiological ligands, the high-affinity binding partner sex hormone binding globulin (SHBG) and the low-affinity partner human serum albumin remain the most important proteins, regulating transport, bioavailability and metabolism of sex steroid hormones [17]. Circulating estrogen in women is mostly produced by the ovaries. In contrast only 20% of circulating estrogen in men is produced in the testes while nearly 80% arises from conversion of testosterone by the aromatase enzyme in local tissue (like skin, adipose tissue, brain, bone) [18]. In women as well as in men the dominant estrogen is 17β-estradiol (E2) [19]. It safeguards new bone formation by promoting osteoblast differentiation [13, 20], inhibiting bone remodelling activation, reducing bone differentiation, restraining osteoclastogenesis and osteoclast survival [21, 22]. The androgen testosterone (T) stimulates proliferation and differentiation of osteoblasts [23] and decreases osteoclast formation in murine cells in vitro [11].

In aging human skeletal bone decreasing levels of estrogen and T are described to be associated with a reduction of bone mineral density and biomechanical properties increasing the fracture risk [15, 24]. The impact of sex steroid hormone deficiency seems to be more pronounced in skeletal rather than in alveolar bone [25, 26], but there is rising evidence that sex steroid hormones also contribute to oral bone homeostasis both in animals and humans [26, 27]. In alveolar bone of androgen depleted rodents, changes of the trabecular bone with increased porosity, resorption, and secretion of pro-inflammatory cytokines have been described [11]. Consequently, hormonal replacement therapy maintained alveolar bone height [26] and increased mandibular bone mineral density in male rats [26]. As estradiol showed a higher impact on maintaining mandibular bone mineral density than T in male rats, different pathways are suggested [28]. So far there is only one human study investigating the association of sex steroid hormones and bone graft regeneration in oral surgery demonstrating a significant positive association of E2 on the rate of NBF after sinus floor augmentation in men [29].

To date there is only minor evidence concerning the influence of sex steroid hormone serum concentration on the capacity of bone graft regeneration in the alveolar bone augmentation. The purpose of the following study was primarily to evaluate the influence of sex steroid hormones on the rate of NBF (primary outcome) and secondarily to evaluate the influence of the body mass index on the NBF rate (secondary outcome) after alveolar ridge augmentation with cortico-cancellous iliac bone grafts. As null hypothesis we assumed no association between sex steroid hormones and NBF rate and no association between BMI and NBF rate.

## Material and methods

The study was approved by the ethics committee of the Charité Medical University of Berlin, Germany, No EA2/089/09. Before enrollment, informed consent was obtained from all individual participants included in the study. None of the patients had known systemic disorders (e.g. diabetes mellitus) that could have affected sex hormone ranges or bone formation rate.

### Inclusion criteria

Patients showing a severe resorption of the jaw with a remaining bone volume of less than 5 mm in height (Cawood Class of jaw atrophy V or VI) underwent onlay grafting of the maxilla and/or the mandible with cortico-cancellous bone from the anterior superior iliac crest. All patients were completely edentulous except two women having partial edentulism. Patients were recruited at the Department of Oral and Maxillofacial-Surgery, Charité-Campus Virchow Clinic, Berlin, Germany. The included women have been gynecologically examined and were identified as postmenopausal.

### Exclusion criteria

Hormone supplementation, periodontitis, diabetes, history of ovariectomy, immunosuppression, cardio-vascular diseases, irradiation and chemotherapy were exclusion criteria. Nicotine users and patients aged < 18 years or participating in other studies were excluded in the present study.

### Flow chart


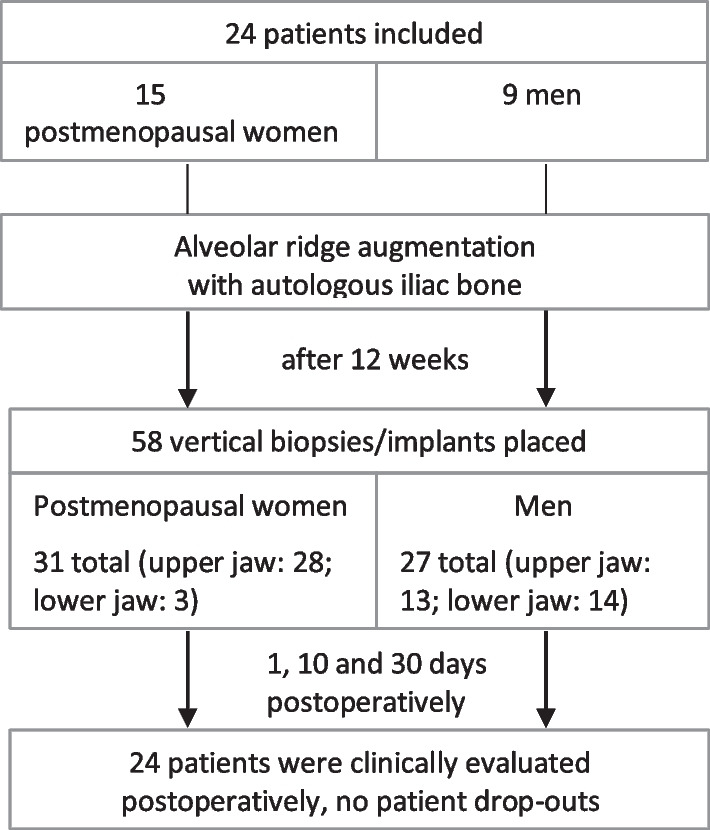


### Grafting procedure

All surgical steps, including bone augmentation and implant placement, were performed under general anesthesia. Iliac graft harvesting was performed as described previously [3]. Patients received intravenous antibiotic (clindamycin 600 mg) during the operation and an oral antibiotic postoperatively (clindamycin 300 mg three times a day) for 7 days. All patients remained hospitalized for 2–3 days. Iliac sutures were removed after 10 days. Clinical evaluation was performed postoperatively at 1, 10 and 30 days to assess complications such as infection, hematoma, postoperative pain, gait disturbance, nerve injury, wound dehiscence, duration of analgesic use and overall acceptance of the grafting procedure.

### Implant placement and trephine biopsies

At the time of implant placement, 12 weeks after grafting [30], vertical biopsies (length: 5–9 mm, diameter: 2 mm) were retrieved from the grafted alveolar ridge at all of the 58 implant positions by using a trephine bur (internal (core) diameter of 2 mm; Straumann AG, Basel, Switzerland). To assure that bone biopsies were taken completely from grafted bone, the same drilling template was used for augmentation and taking vertical bone biopsies. A total of 58 implants were placed according to the manufacturers surgical protocol: 54 Camlog Root-Line implants (Camlog Biotechnologies AG, Basel, Switzerland) and four Straumann Tissue Level implants (Straumann AG, Basel, Switzerland). The mobilized mucoperiosteal flap was repositioned and sutured using running as well as interrupted sutures (5–0 Monocryl, Ethicon). Unloaded healing time for implants was 12 weeks in the maxilla and 6 weeks in the mandible. At loading primary stability (> 30 Ncm) of the implants was monitored using a torque ratchet.

### Histomorphometric analysis

After removal, the specimen’s coronal side was marked with ink (Marker II SuperFrost, Precision Dynamics Corporation, San Fernando, California, USA) allowing to distinguish the coronal and apical region. Subsequent bone biopsies were fixed in 4% formalin for 48 h and subsequently decalcified in 17% nitric acid for 24 h. Specimens were embedded in paraffin, sliced into 5 µm thick serial sections (Leica Jung Supercut Model 2065, Leica Microsystems GmbH, Wetzlar, Germany) and stained with Masson-Goldner`s trichrome (Kit 3459.1, Carl Roth GmbH + Co. KG, Karlsruhe, Germany).

Biopsies were further examined by placing a square, defined as an area of 2500 µm^2^, as *region of interest* (ROI) in the coronal and apical portion of each specimen. Histomorphometric quantification of new bone formation was performed by a single experienced observer who was blinded to the clinical data using digital high-resolution light microscopy (AxioCam HRc, Carl Zeiss AG, Oberkochen, Germany) combined with analySIS FIVE image analysis software (Olympus Deutschland GmbH, Hamburg, Germany). Calibration was carried out by placing a stage micrometer 25 + 50/10 mm (Zeiss, Göttingen, Germany) diagonally across the image. Newly formed bone was put in relation to total bone in each specimen prior to analysis. New bone and mature bone were distinguished by color (turquois-green versus red-brown), morphology (woven bone versus lamellar bone) and present cell types like previously described [2]. Each patient received at least two implants, the rate of NBF of each specimen was summed and averaged per patient. Values for the rate of NBF are always adjusted to the coronal and apical area unless described otherwise.

### Serum quantification 17ß-estradiol, testosterone and SHBG

At the day of the grafting procedure, venous blood samples (5 ml each) were collected preoperatively from 8:00 to 10:00 am after an overnight fast in order to minimize circadian variation in sex steroid hormone serum levels. Sex steroid hormones were extracted from serum by centrifugation at 2.500 × g for 10 min (Labofuge 200, Thermo Scientific, Heraeus Holding GmbH, Hanau, Germany) and subsequently analyzed by an endocrine research laboratory (Labor Berlin Kompetenz Charité and Vivantes GmbH, Berlin, Germany). Serum levels of E2 and SHBG were quantified by electrochemiluminescence immunoassay (Roche AG, Basel, Switzerland), while T was measured by radioimmunoassay (Siemens Inc. Munich, Germany). Reference ranges can be found in supplement 1 (online resource).

### BMI measurement

Weight and height were measured to the nearest 0.1 kg and 0.1 cm respectively and *body mass index* (BMI) was calculated as the ratio of weight to height squared [kg/m^2^]. Reference ranges can be found in supplement 2 (online resource).

### Statistical analysis

For statistical analysis, mean, standard deviation and range were computed. Scatter plots were used for graphical presentation, thus mean values per patient were used. To take clustering due to several measurements into account linear mixed models were applied to test for differences between the groups.

To analyze the influence of 17ß-E2, T and SHBG on the rate of NBF, the variables gender, age and sample site (coronal/apical) were used to adjust. Additional subgroup analysis for men and women was done.

To analyze the influence of BMI on rate of NBF, the mean values per patient were used in a linear regression model adjusting for gender. Statistical analysis was performed using STATA (StataCorp LT, College Station, TX, Version 17.0).

## Results

**Fig. 1:**
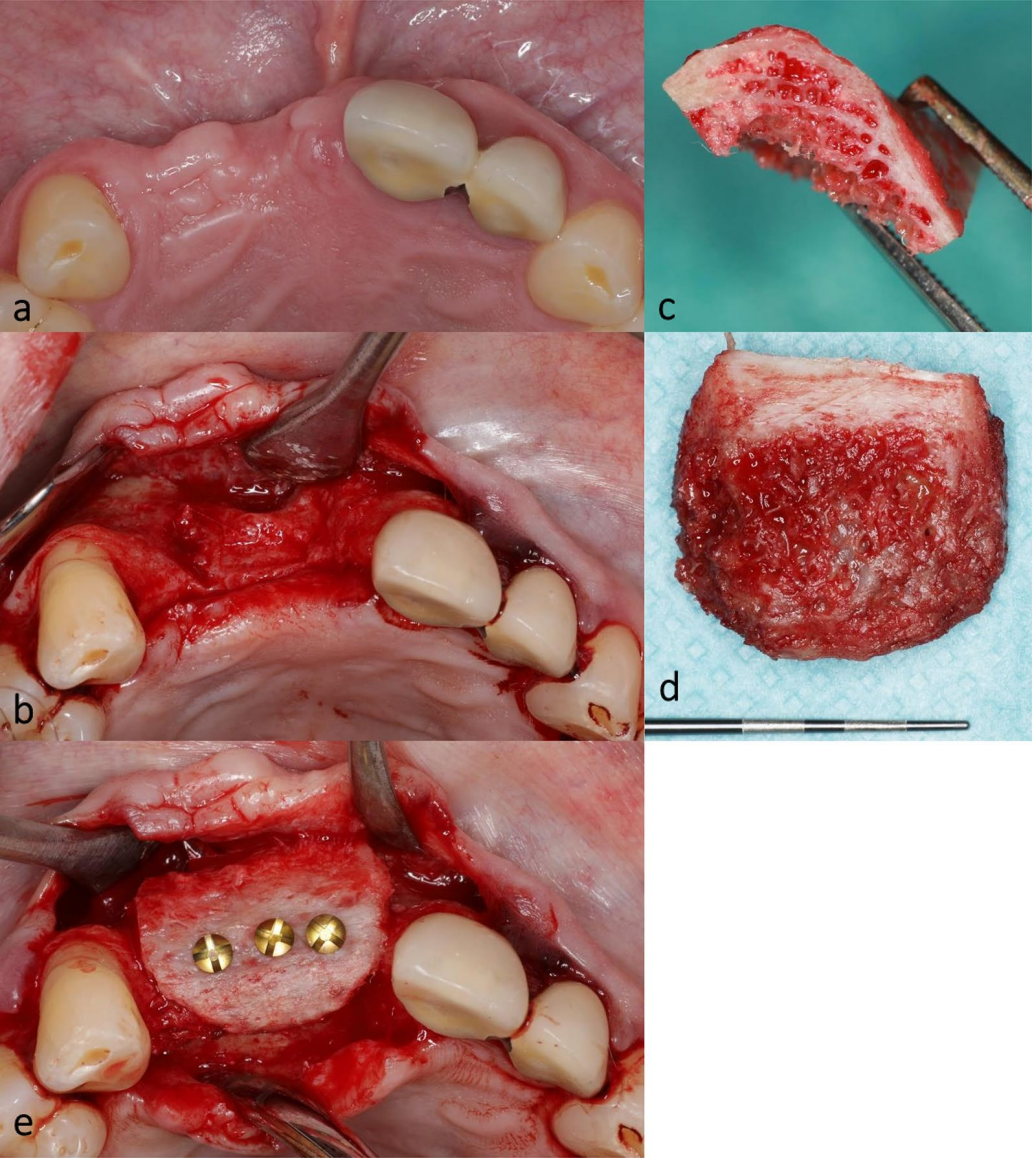
Clinical pictures of the alveolar ridge augmentation procedure with autologous cortico-cancellous bone from the iliac crest. **a** clinical situation before augmentation; **b** atrophic alveolar ridge; **c** autologous cortico-cancellous bone harvested from the anterior superior iliac crest; **d** iliac bone graft adapted on alveolar ridge defect; **e** bone graft fixated at recipient bone

Twenty-four patients (15 females and 9 males) with a mean age of 57.8 years received onlay grafting of the maxilla and/or the mandible with cortico-cancellous bone from the anterior superior iliac crest (Table 1; Fig. 1). Graft healing was uneventful, no dehiscence was observed. At the time of implant placement, 12 weeks after grafting, 58 biopsies (31 maxilla, 27 mandible) were obtained. Subsequently all planned implants were successfully inserted. Within a 30-day follow-up there were no postoperative complications such as pain, hematoma formation, infection, nerve injury or wound dehiscence. None of the included patients dropped out of the study.

**Table 1:** Age, BMI, concentration of 17β-estradiol, testosterone and sex hormone binding globulin (SHBG) of the included patients

		Age [years]	BMI [kg/m^2^]	Estradiol [pmol/l]	Testosterone [nmol/l]	SHBG [nmol/l]
Female (*n* = 15)	Mean ± SD	58.7 ± 7.5	24.3 ± 3.3	38.5 ± 39.7	0.5 ± 0.3	75.3 ± 41.5
	Range	42–68	18.8–29.3	18.0–166.0	0.1–1	32.0–185.1
Male (*n* = 9)	Mean ± SD	56.3 ± 10.8	23.5 ± 2.9	108.3 ± 46.5	14.6 ± 7.3	62.5 ± 31.4
	Range	39–71	19.8–28.0	35.0–157.0	2.7–24.4	22.0–111.5
Total (*n* = 24)	Mean ± SD	57.8 ± 8.8	24.0 ± 3.1	64.7 ± 53.9	5.8 ± 8.2	70.5 ± 37.8
	Range	39–71	18.8–29.3	18.0–166.0	0.1–24.4	22.0–185.1

*SD* standard deviation

Blood screening revealed a significantly higher (*p* < 0.01) E2 serum concentration in men, than in postmenopausal women, as well as a significantly higher (*p* < 0.001) T serum concentration in men than in postmenopausal women (Table 1). Regardless of gender SHBG serum concentration showed interindividual differences among all patients (Table 1).

Histomorphometric analysis revealed an overall mean new bone formation of 32.5% (range: 3.2–81.7%) in a total of 58 biopsies. In males the mean rate of NBF of 38.1% was significantly higher (*p* < 0.01) than in females with a mean of 27.6% (Table 2). Independent of sex, the rate of NBF in the apical area significantly (*p* < 0.0001) exceeded the rate of NBF in the coronal area. There was no significant difference in the amount of NBF between the maxilla and the mandible in females (*p* = 0.083), as well as in males (*p* = 0.546). Independent of gender, jaw and coronal vs. apical site interindividual differences (SD of 7.3) have been detected. Age and rate of NBF were negatively (regression coefficient: -0.24) associated, although not significantly (*p* = 0.353).

**Table 2:** Gender, sample area, mean, standard deviation and range of new bone formation of the included patients

		New bone formation rate [in %]	
		Mean ± SD	Range
Female	Apical	34.0 ± 13.8	10.8–77.1
	Coronal	21.2 ± 12.2	3.2–51.2
	Total	27.6 ± 14.5	3.2–77.1
Male	Apical	46.1 ± 18.6	4.6–81.7
	Coronal	30.1 ± 15.1	5–72.9
	Total	38.1 ± 18.6	4.6–81.7
Total	Apical	39.7 ± 17.2	4.6–81.7
	Coronal	25.3 ± 14.3	3.2–72.9
	Total	32.5 ± 17.3	3.2–81.7

*SD standard deviation*

### Association of 17β-estradiol, testosterone, SHBG with the rate of new bone formation

**Fig. 2:**
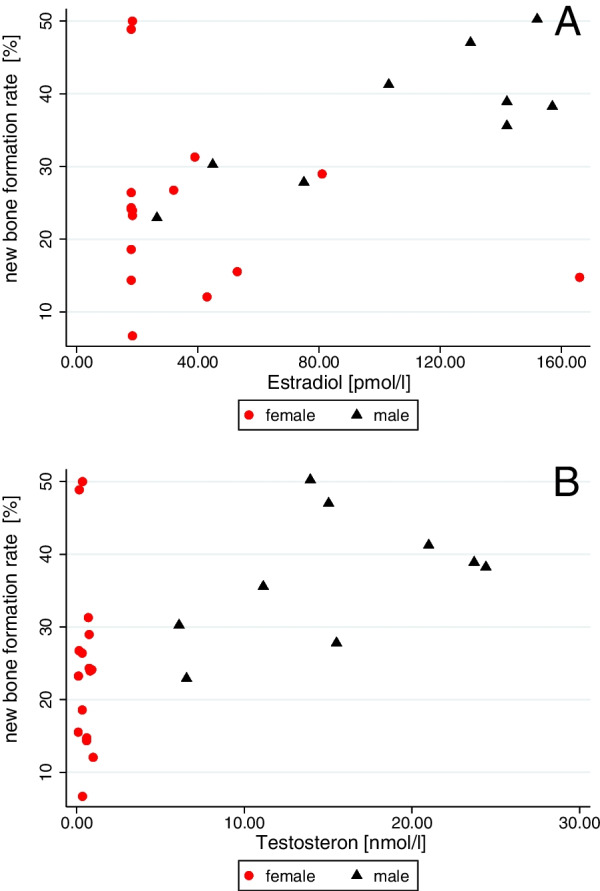
Association of sex steroid hormones and new bone formation rate. While there is a significant association of **A** 17β-estradiol [pmol/l] and new bone formation rate [%] in men (men: regression coefficient (rc) = 0.128, *p* = 0.020; women: rc = − 0.088, *p* = 0.246), there is **B** no significant association of testosterone [nmol/l] and new bone formation rate [%] detectable (men: rc = 0.690, *p* = 0.110; women: rc = − 11.499, *p* = 0.257) after adjustment for age, gender and coronal vs. apical site

There was a positive association between E2 (regression coefficient (rc) = 0.032, *p* = 0.533) and T (rc = 0.713, *p* = 0.180) with overall rate of NBF (Fig. 2). After adjustment for age and coronal vs. apical site a significant association to the rate of NBF was only seen for E2 in men (rc = 0.128, *p* = 0.020). E2 in women (rc = − 0.088, *p* = 0.246), and T in both genders, as well as the amount of SHBG (rc = − 0.001, *p* = 0.897) were not associated.

### Influence of Body mass index on new bone formation rate

**Fig. 3:**
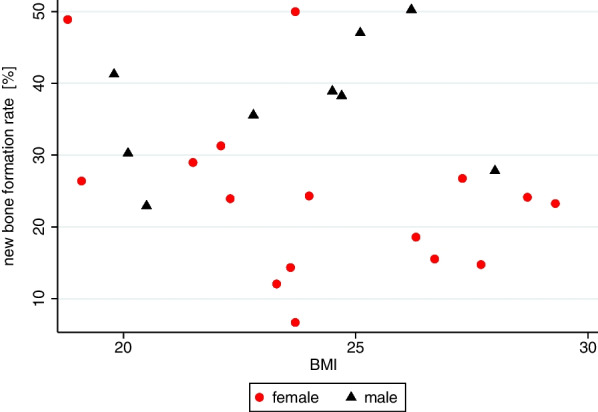
Association of BMI and new bone formation rate. There is no significant association of *body mass index* (BMI) with new bone formation rate in men (regression coefficient (rc) = 0.956, *p* = 0.413) as well as in women (rc = − 1.479, *p* = 0.129) detectable

Fifteen patients with normal weight (9 women and 6 men) and nine overweight patients (6 women and 3 men) with a mean BMI of 24.0 received iliac bone grafts (Table 1). Regardless of gender a negative association between BMI and rate of NBF was detected (Fig. 3). However, the observed association was not significant (rc = − 0.957, *p* = 0.248).

## Discussion

The influence of sex steroid hormones on the rate of NBF after two-stage alveolar ridge augmentation with autologous cortico-cancellous iliac bone grafts has not been investigated to date. In the present study the rate of NBF was determined by histomorphometric measurement and associated to serum levels of E2 and T.

The overall mean rate of NBF in the present study was within the reported range of 33–46% for iliac onlay grafts in previous studies [2, 3] with a broad range of new bone formation (3.2–81.7%) within the same healing time [2, 3], which is in accordance with current findings suggesting interindividual differences in human bone biology [31]. In the current study the rate of NBF in men was significantly higher than in women. This is consistent with data from a study regarding sinus floor augmentation using alloplastic β-tricalcium phosphate (β-TCP) in which men had a tendency of a higher rate of new bone formation compared to women [29]. Within this study the site of augmentation (maxilla vs. mandible) had no influence on the rate of NBF. This is in line with findings of studies regarding the amount of new bone formation in human alveolar extraction sockets [32]. Previous studies evaluating the rate of new bone formation in iliac onlay grafts comprised only the maxilla [2, 3]. Apical new bone formation is significantly higher compared to the coronal site, as previously reported [3]. These insights support the assumption that bone graft revascularization is initiated from the recipient bone [33].

Age and rate of NBF was negatively associated in our data. This is in accordance with previous studies using cancellous freeze-dried bone block allografts or alloplastic bone substitute material for grafting of the maxilla [29, 34, 35]. In contrast to Nissan et al. [34] the detected association between NBF rate and age, was not significant in the study of Knabe et al. [29], even though they included not only a larger number of patients (*n* = 120), but also a cohort with a broader age-distribution. In a study evaluating the bone regeneration after extraction of a tooth a negative association between angiogenesis (CD31 positive cells) and age was observed [32]. Data showing a significant difference in the expression of osteogenic markers (osteocalcin, bone sialoprotein, alkaline phosphatase) in patients under 50 years compared to patients aged over 50 [29] after sinus floor augmentation, should not be compared directly to the amount of NBF. The osteogenic markers reveal the stage of the osteogenic cell, but no association to the ability of bone formation can be drawn. However, there is rising evidence that decreasing NBF rate with age might be based on age-dependent proteome changes [36].

In this study, association of the NBF rate with the BMI in regard to gender was not possible due to the limited number of patients within the category normal weight and overweight and no obese or underweight patients being included. Comparable to our results, Knabe et al. [29], who also included obese patients, could not find a significant association between BMI and the rate of NBF after sinus floor augmentation with ß-TCP in females. For males they demonstrated a significant positive association between BMI and the rate of NBF [29]. This is in line with findings associating a higher BMI with a higher bone mineral density and a reduced risk of fracture in skeletal bone [37]. However, emerging evidence indicates a more complex association of BMI and bone health [38], as association between body fat mass and bone mineral density, as well as a relationship between BMI and fracture risk were shown to depend on anatomical sites [39]. Because higher body fat mass leads to crucial metabolic adjustments like an upregulated secretion of various hormones involved in metabolic regulation (like insulin, amylin and leptin), it is associated with an increased rate of aromatization of androgens to estrogens in adipose tissue [40, 41], as well as with reduced SHBG serum concentration leading to an increased fraction of free E2 and free T in blood [42]. In summary, the interaction of obesity with bone metabolism and bone microarchitecture is complex and is still not fully understood [43]. In this context BMI has to be considered with caution, as it does not include body fat percentage and body fat distribution, which is known to affect bone density as well as bone microarchitecture [43].

In accordance to our results Knabe et al. [29] revealed a significant positive association between the NBF rate and E2 in men, but not in women. The group of females evaluated was of inhomogeneous composition, consisting not only of postmenopausal women like in our study, but also of premenopausal women, females with history of ovariectomy and hormone substituted females [29].

Concerning Testosterone, our data revealed no significant association between total T and the rate of NBF in both women and men. In the study of Knabe et al. [29] the free androgen index, defined by dividing total T multiplied with an association constant through SHBG was used, showing no significant association between the NBF rate and T in men but conflicting results in women. Depending on the utilized statistical model, women either showed no association (using Spearman analysis) or a significant negative association (using linear regression analysis) concerning the NBF rate and T [29]. As measurement of free sex steroid hormone concentration are complex and time-consuming, calculation of free sex steroid hormones, using measured total E2/total T and SHBG in conjunction with equilibrium-binding theory or empirical equations became popular, regardless of their unreliability [44, 45]. Equilibrium-binding equation-based calculation methods presented to be even worse than assumption-free empirical formulae [44, 46], as the SHBG as well as the albumin association constant for E2/T differs up to 14-fold between different authors [16, 47, 48]. Therefore, results based on the calculated free androgen index should be considered with caution. As total E2/total T (determined by direct radioimmunoassay) were shown to associate strongly with free E2/free T (measured by dialysis) in postmenopausal women [49], our results may give at least a hint at the impact of bioavailable sex steroid hormones on bone regeneration capacity in the alveolar ridge. Additionally, it has to be kept in mind, that effects of E2 and T might not be fully separable from each other, as T is partially aromatized into E2 [50]. Further, variations in number, density and distribution of sex steroid hormone receptors in the alveolar ridge may affect the influence of sex steroid hormones on bone graft regeneration in the human alveolar ridge. Thus, further research investigating the association of the NBF rate in relation to jaw region could be helpful proving aforementioned theory.

In our study SHBG did not show any influence on the NBF rate in both genders. As SHBG concentration is known to increase with age [51], investigation of SHBG influencing bone regeneration capacity in a patient cohort with a broader age distribution might be more relevant.

Based on the results of this study it can be concluded that oral bone graft regeneration might depend on sex steroid hormone serum levels in humans. Nevertheless, we have to acknowledge several limitations. We only included postmenopausal women, as our study focused on patients receiving iliac onlay grafts, used for critical size defects, which mainly occur in older people. As premenopausal women exhibit a higher ratio of E2 to T, premenopausal women should be included in future studies. Thereby blood sampling should be scheduled for the beginning of the female cycle in order to avoid misinterpretation because E2 concentration is known to fluctuate during the female cycle [52].

Since this study was conceived as a pilot study, the included patient number is rather low. To obtain an adequate power for the investigation of one observation per patient further studies should include a minimum of 200 patients or rather 45 patients (each one third premenopausal, postmenopausal women and men) to receive significant results (5% significance level and 80% power) concerning the rate of NBF and E2 or NBF rate and T, respectively. Besides a homogeneous patient cohort also the normalization of host factors among the included patients is important for a thorough understanding concerning the association of sex steroid hormones in bone regeneration.

Total E2, T and SHBG, as well as free E2 and T should be assessed in future studies, using valid measurement methods to determine the physiological relevance of sex steroid hormone serum concentration on bone regeneration capacity. From a practical point of view measurement of salivary E2/T by radioimmunoassay, enzyme-linked immunosorbent assay or LC-MS/MS [40] might be conceivable in future oral health studies as it seems to associate strongly with free serum E2 and free serum T [53]. In addition, serum concentration of estrone, which is considered to be a weak estrogen due to its low binding affinity to the estrogen receptor, might be relevant in future investigations, as it is the primary sex steroid hormone in postmenopausal women [54].

In summary we provide evidence for a positive association between 17β-estradiol or rather testosterone and new bone formation rate in the human jaw independent of gender. Further studies are necessary to confirm these findings under consideration of total estradiol, total testosterone, SHBG, free estradiol, free testosterone and estrone in a homogeneous patient cohort. Such knowledge might enable future clinicians to improve treatment modality for optimal bone regeneration capacity in the human jaw.

## Conclusions

Based on the histomorphometric analysis of iliac bone grafts, gender specific differences were recorded showing a significantly higher rate of new bone formation in men compared to postmenopausal women with a similar age distribution. SHBG serum levels had no influence on new bone formation rate. There was no significant association between new bone formation rate and age or new bone formation rate and BMI. A positive association between estradiol as well as testosterone with the rate of NBF indicate a role of sex steroid hormones in alveolar bone regeneration, although the observed influence was only significant for estradiol in men.

## Supplementary Information

**Additional file 1. Supplement 1.** Body mass index (BMI), 17β-estradiol, testosterone, sex hormone binding globulin and meannew bone formation (NBF) in female and male patients. **Supplement 2**. Reference ranges (**a**) of the *body mass index* (BMI), according to the *World HealthOrganization* (WHO, 2000), regardless of gender and (**b**) of the sex steroid hormone serumconcentrations according to the criteria of the Labor Berlin - Charité Vivantes GmbH.

## Data Availability

All data generated or analyzed during this study are included in this published article and its supplementary information files.

## Abbreviations

BMI: Body mass index
E2: 17β-Estradiol
NBF: New bone formation
Rc: Regression coefficient
SHBG: Sex hormone binding globulin
T: Testosterone
