# Supplementary material for: Association of sex steroid hormones and new bone formation rate after iliac onlay grafting: a prospective clinical pilot study
Source: Int J Implant Dent. 2022 Nov 15;8:53. doi: 10.1186/s40729-022-00447-x (PMC9663769; doi:10.1186/s40729-022-00447-x)
Supplement: Supplementary file 1 — Additional file 1. Supplement 1. Body mass index (BMI), 17β-estradiol, testosterone, sex hormone binding globulin and meannew bone formation (NBF) in female and male patients. Supplement 2. Reference ranges (a) of the body mass index (BMI), according to the World HealthOrganization (WHO, 2000), regardless of gender and (b) of the sex steroid hormone serumconcentrations according to the criteria of the Labor Berlin - Charité Vivantes GmbH. [file 40729_2022_447_MOESM1_ESM.docx]

**Association of sex steroid hormones and new bone formation rate after iliac onlay grafting: a prospective clinical pilot study**

Victoria Constanze Landwehr^1^, Tobias Fretwurst^1^, Julia Heinen^2^, Kirstin Vach^3^, Katja Nelson^1^, Susanne Nahles^2^, Gerhard Iglhaut^1^

^1^ Department of Oral and Maxillofacial Surgery, Center for Dental Medicine, Medical Center – University of Freiburg, Faculty of Medicine – University of Freiburg, Freiburg, Germany
^2^ Department of Oral and Maxillofacial Surgery, Berlin Institute of Health, Corporate Member of Freie Universität Berlin, Charité – Universitätsmedizin Berlin, Humboldt-Universität zu Berlin, Berlin, Germany

^3​^ Institute of Medical Biometry and Statistics, Faculty of Medicine and Medical Center, University of Freiburg, Freiburg, Germany

**Adresses:**

Victoria Constanze Landwehr, Tobias Fretwurst, Katja Nelson and Gerhard Iglhaut

Department of Oral and Maxillofacial Surgery

Center for Dental Medicine

Hugstetter Straße 55

79106 Freiburg

Germany

Julia Heinen and Susanne Nahles

Department of Oral and Maxillofacial Surgery

Charité– Universitätsmedizin Berlin

Augustenburger Platz 1
13353 Berlin

Germany

Kirstin Vach

Institute of Medical Biometry and Statistics

Faculty of Medicine and Medical Center

Hebelstraße 11

79104 Freiburg

Germany

**Corresponding author:**

Victoria Constanze Landwehr

Department of Oral and Maxillofacial Surgery

Center for Dental Medicine

Medical Center – University of Freiburg

Faculty of Medicine – University of Freiburg

Freiburg – Germany

Telephone: 0049-761-270-49820

Fax: 0049-761-270-48010

Email: victoria.landwehr@uniklinik-freiburg.de

**Supplement 1**

Body mass index (BMI), 17β-estradiol, testosterone, sex hormone binding globulin and mean new bone formation (NBF) in female and male patients.


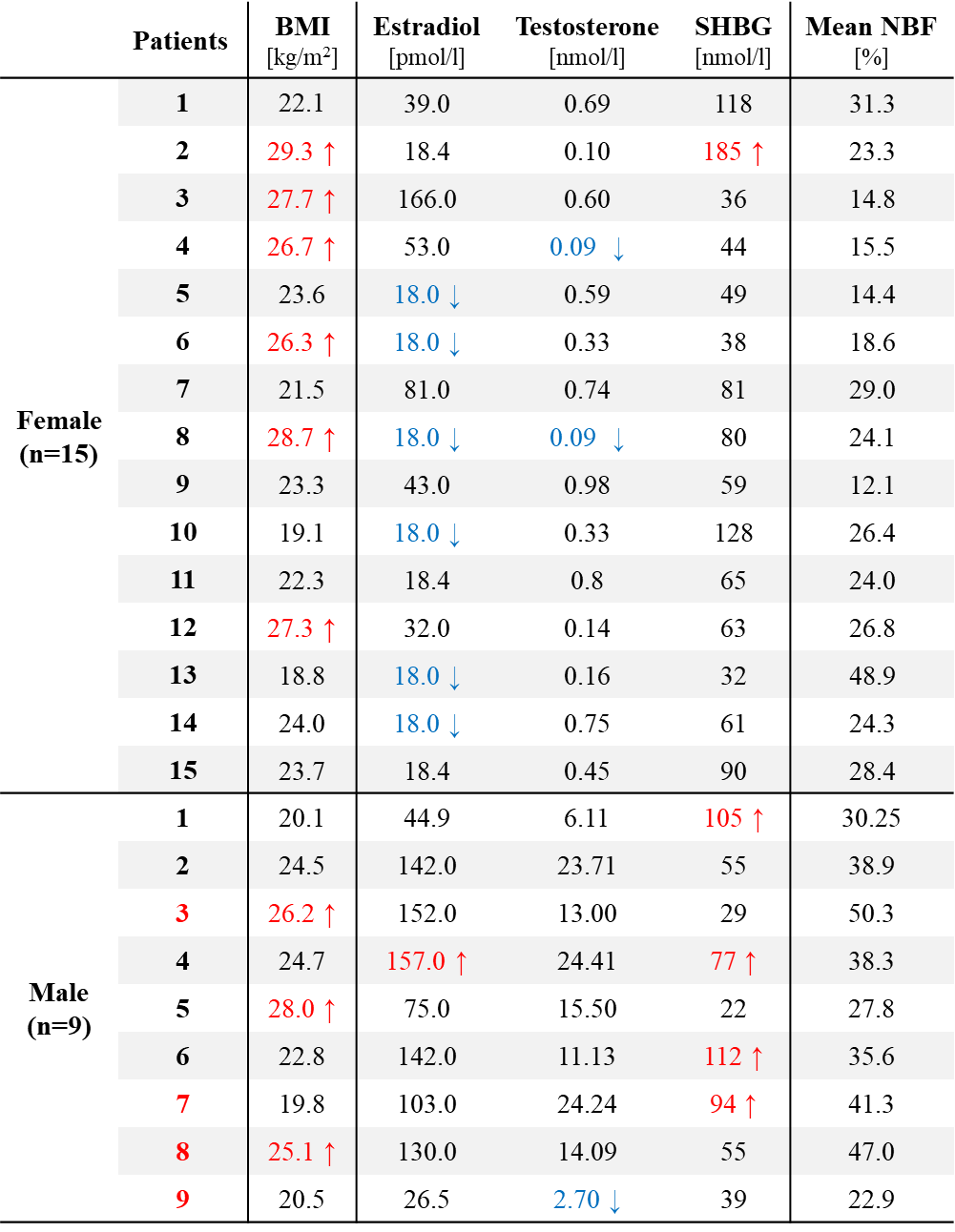


Values above the reference range and overweighted patients are colored red and marked with ↑, values below the reference range are colored blue and marked with ↓.

**Supplement 2**

Reference ranges (a) of the *body mass index* (BMI), according to the *World Health Organization* (WHO, 2000), regardless of gender and (b) of the sex steroid hormone serum concentrations according to the criteria of the Labor Berlin - Charité Vivantes GmbH.

| **Classification** | **BMI** |
| --- | --- |
| Underweight | < 18.5 |
| Normal weight | 18.5 - 24.9 |
| Overweight or pre-obesity | 25 - 29.9 |
| Obesity | ≥ 30 |

**a**

| **Gender** | **Estradiol serum concentration [pmol/l]** |
| --- | --- |
| Female (postmenopausal) | 18.4 - 201 |
| Men (> 21 years) | 28 - 156 |

| **Gender** | **Testosterone serum concentration [nmol/l]** |
| --- | --- |
| Female (postmenopausal) | 0.1 - 1.6 |
| Men (20 - 49 years) | 7.6 - 31.4 |
| Men (> 50 years) | 4.6 - 30.9 |

| **Gender** | **SHBG serum concentration [nmol/l]** |
| --- | --- |
| Female (postmenopausal) | 14.4 - 136 |
| Men (13 - 49 years) | 10.6 - 72.3 |
| Men (> 50 years) | 14.2 - 78.0 |

**b**
